# Supplementary figures and images for: Iterative segmentation and classification for enhanced crop disease diagnosis using optimized hybrid U-Nets model (part 2 of 2)
Source: PeerJ Comput Sci. 2025 Jun 11;11:e2543. doi: 10.7717/peerj-cs.2543 (PMC12190645; doi:10.7717/peerj-cs.2543)

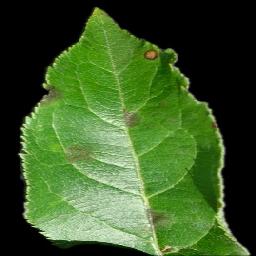

Supplement: Supplemental Information 2 [file peerj-cs-11-2543-s002.zip › Apple/Apple___Apple_scab/2bfd8ee0-af6b-42fd-ae61-fc5e92ec1b67___FREC_Scab 2988_final_masked.jpg]

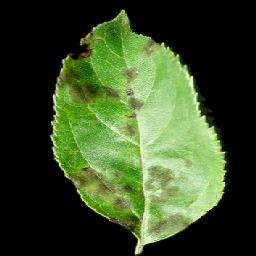

Supplement: Supplemental Information 2 [file peerj-cs-11-2543-s002.zip › Apple/Apple___Apple_scab/2c648fcd-d637-4559-beaa-9dd6079646fb___FREC_Scab 3213_final_masked.jpg]

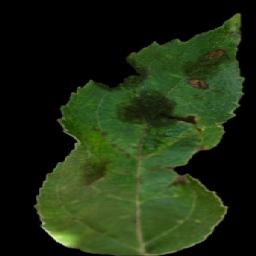

Supplement: Supplemental Information 2 [file peerj-cs-11-2543-s002.zip › Apple/Apple___Apple_scab/2c7ca7c8-1a6c-4811-b9b0-b054c11a5397___FREC_Scab 3260_final_masked.jpg]

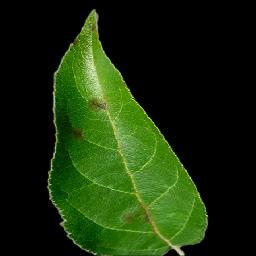

Supplement: Supplemental Information 2 [file peerj-cs-11-2543-s002.zip › Apple/Apple___Apple_scab/2c89ceaf-748c-4371-80d0-d01855f04a92___FREC_Scab 2962_final_masked.jpg]

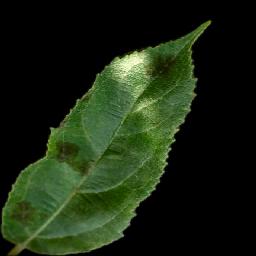

Supplement: Supplemental Information 2 [file peerj-cs-11-2543-s002.zip › Apple/Apple___Apple_scab/2d329823-7ad5-4189-89b7-4bdf24f9c393___FREC_Scab 3507_final_masked.jpg]

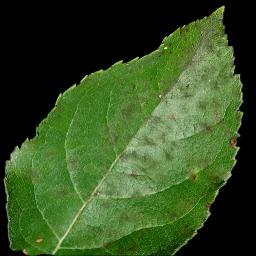

Supplement: Supplemental Information 2 [file peerj-cs-11-2543-s002.zip › Apple/Apple___Apple_scab/2d4c8a12-a34c-4c8b-b357-271d87532ece___FREC_Scab 3090_final_masked.jpg]

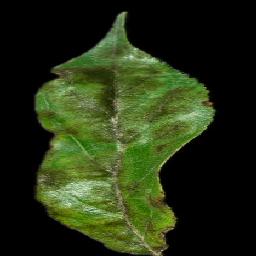

Supplement: Supplemental Information 2 [file peerj-cs-11-2543-s002.zip › Apple/Apple___Apple_scab/2d5265d2-be99-4e9f-a085-d309cc01b751___FREC_Scab 3023_final_masked.jpg]

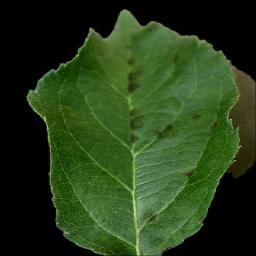

Supplement: Supplemental Information 2 [file peerj-cs-11-2543-s002.zip › Apple/Apple___Apple_scab/2dd8b8f8-46b0-4bc6-b817-ea7163f4b28b___FREC_Scab 3473_final_masked.jpg]

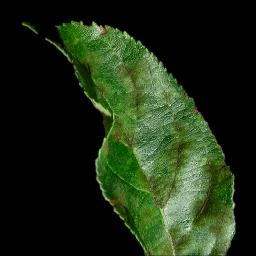

Supplement: Supplemental Information 2 [file peerj-cs-11-2543-s002.zip › Apple/Apple___Apple_scab/2e264ee0-7679-4ad2-a19a-1ac4d2e84079___FREC_Scab 2984_final_masked.jpg]

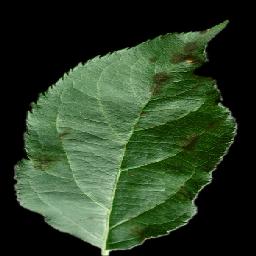

Supplement: Supplemental Information 2 [file peerj-cs-11-2543-s002.zip › Apple/Apple___Apple_scab/2f1e68c0-c059-46fd-bbf3-f160817abdc3___FREC_Scab 3535_final_masked.jpg]

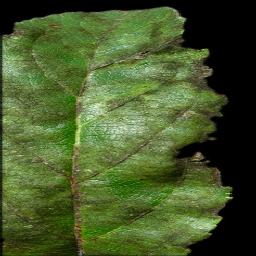

Supplement: Supplemental Information 2 [file peerj-cs-11-2543-s002.zip › Apple/Apple___Apple_scab/2f668a95-80ab-40ac-98ef-657bd85b668b___FREC_Scab 3247_final_masked.jpg]

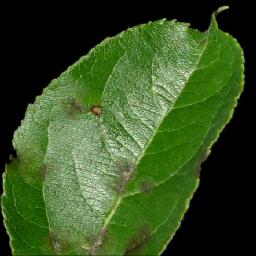

Supplement: Supplemental Information 2 [file peerj-cs-11-2543-s002.zip › Apple/Apple___Apple_scab/30111fee-0c22-4ca4-aeb4-ac14a9a5d651___FREC_Scab 3097_final_masked.jpg]

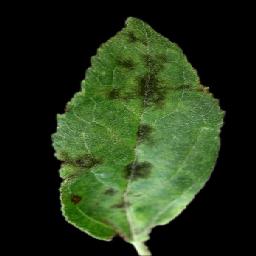

Supplement: Supplemental Information 2 [file peerj-cs-11-2543-s002.zip › Apple/Apple___Apple_scab/320dff9e-6d59-4bec-9e7c-930444343e3b___FREC_Scab 3398_final_masked.jpg]

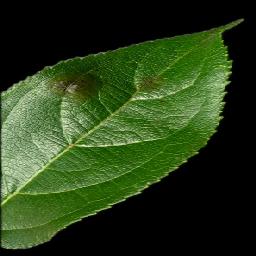

Supplement: Supplemental Information 2 [file peerj-cs-11-2543-s002.zip › Apple/Apple___Apple_scab/321c7135-853f-444d-9952-a893e660d966___FREC_Scab 2958_final_masked.jpg]

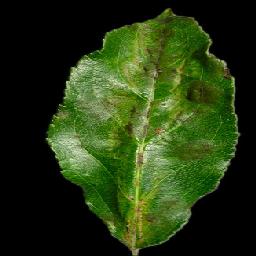

Supplement: Supplemental Information 2 [file peerj-cs-11-2543-s002.zip › Apple/Apple___Apple_scab/32454154-edbf-464f-8097-e99a3d9a15af___FREC_Scab 2936_final_masked.jpg]

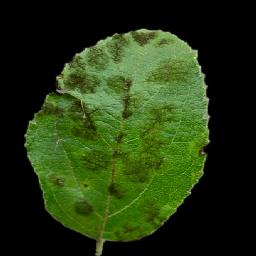

Supplement: Supplemental Information 2 [file peerj-cs-11-2543-s002.zip › Apple/Apple___Apple_scab/32550bbb-cdf1-4805-8167-27dc5a8e87d2___FREC_Scab 3195_final_masked.jpg]

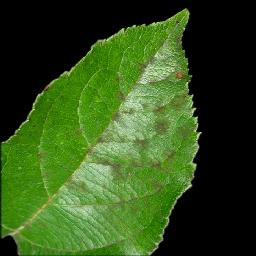

Supplement: Supplemental Information 2 [file peerj-cs-11-2543-s002.zip › Apple/Apple___Apple_scab/334f8e1a-5989-476e-88f4-8a45c304e12c___FREC_Scab 3035_final_masked.jpg]

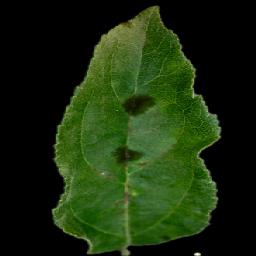

Supplement: Supplemental Information 2 [file peerj-cs-11-2543-s002.zip › Apple/Apple___Apple_scab/33ebf0dd-f971-4f11-aa93-45df7d55a9ea___FREC_Scab 3182_final_masked.jpg]

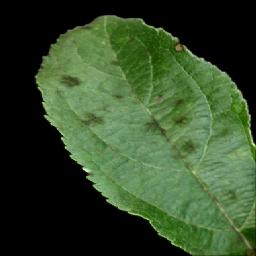

Supplement: Supplemental Information 2 [file peerj-cs-11-2543-s002.zip › Apple/Apple___Apple_scab/34c2823b-d1c5-4bd8-a5a7-74bdef959e10___FREC_Scab 3484_final_masked.jpg]

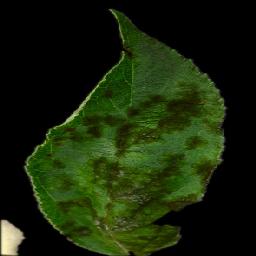

Supplement: Supplemental Information 2 [file peerj-cs-11-2543-s002.zip › Apple/Apple___Apple_scab/34c37353-8429-499e-bd38-ac7501f79e90___FREC_Scab 3501_final_masked.jpg]

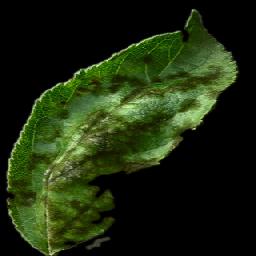

Supplement: Supplemental Information 2 [file peerj-cs-11-2543-s002.zip › Apple/Apple___Apple_scab/3523d2d8-0da9-4b83-b09d-21bd96860a3d___FREC_Scab 3499_final_masked.jpg]

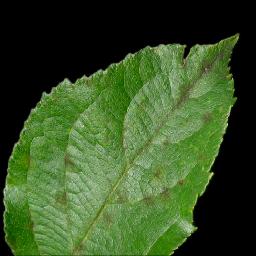

Supplement: Supplemental Information 2 [file peerj-cs-11-2543-s002.zip › Apple/Apple___Apple_scab/352a5659-3552-4e94-8333-c3715d38cc27___FREC_Scab 3049_final_masked.jpg]

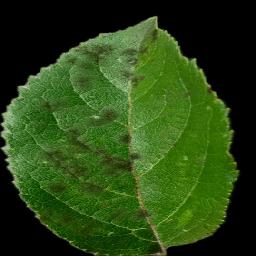

Supplement: Supplemental Information 2 [file peerj-cs-11-2543-s002.zip › Apple/Apple___Apple_scab/35694cb9-efe9-441a-a810-13e065de7925___FREC_Scab 3320_final_masked.jpg]

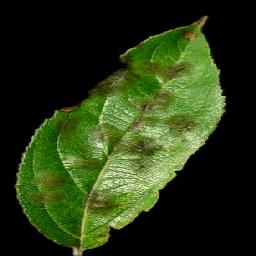

Supplement: Supplemental Information 2 [file peerj-cs-11-2543-s002.zip › Apple/Apple___Apple_scab/35fde58e-46ea-4d1b-9580-ead7a2d0cb3b___FREC_Scab 2952_final_masked.jpg]

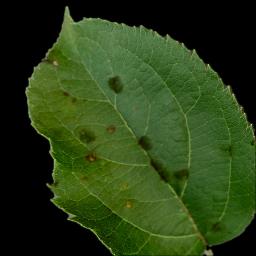

Supplement: Supplemental Information 2 [file peerj-cs-11-2543-s002.zip › Apple/Apple___Apple_scab/3636b2d7-b888-437b-b4e3-a8af4bb62763___FREC_Scab 3148_final_masked.jpg]

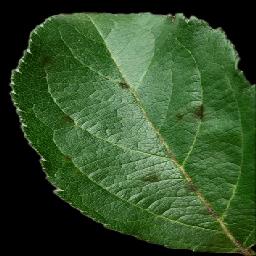

Supplement: Supplemental Information 2 [file peerj-cs-11-2543-s002.zip › Apple/Apple___Apple_scab/3652fd23-ba4c-4958-8db8-3aff8f614dfd___FREC_Scab 3377_final_masked.jpg]

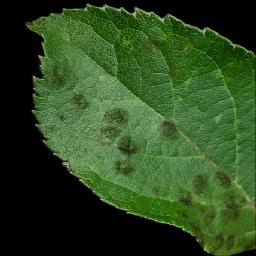

Supplement: Supplemental Information 2 [file peerj-cs-11-2543-s002.zip › Apple/Apple___Apple_scab/36d99a07-4a43-4416-b204-6886f71d5039___FREC_Scab 3294_final_masked.jpg]

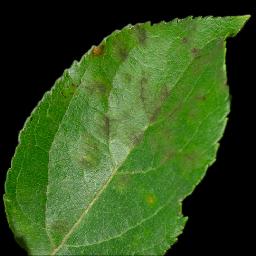

Supplement: Supplemental Information 2 [file peerj-cs-11-2543-s002.zip › Apple/Apple___Apple_scab/37659856-0fc8-430b-871a-af4f0b557e49___FREC_Scab 3081_final_masked.jpg]

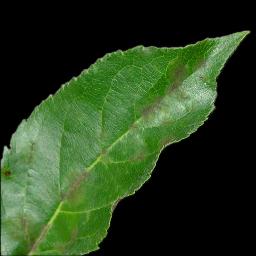

Supplement: Supplemental Information 2 [file peerj-cs-11-2543-s002.zip › Apple/Apple___Apple_scab/376aaef3-8649-43aa-89f8-166bd349a3d3___FREC_Scab 3064_final_masked.jpg]

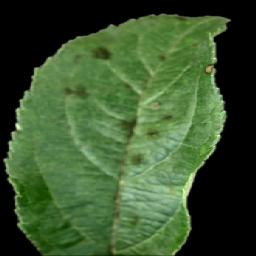

Supplement: Supplemental Information 2 [file peerj-cs-11-2543-s002.zip › Apple/Apple___Apple_scab/38e76f0a-63cb-460a-b0fa-f20e561dc2c0___FREC_Scab 3481_final_masked.jpg]

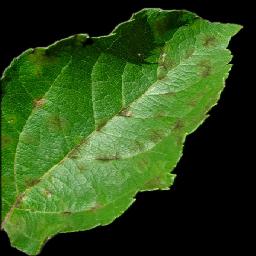

Supplement: Supplemental Information 2 [file peerj-cs-11-2543-s002.zip › Apple/Apple___Apple_scab/3bf2f5f8-4578-405a-88c6-c5338620a078___FREC_Scab 3059_final_masked.jpg]

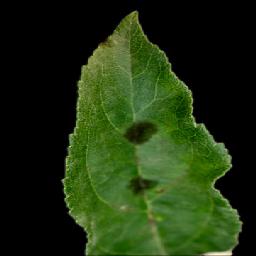

Supplement: Supplemental Information 2 [file peerj-cs-11-2543-s002.zip › Apple/Apple___Apple_scab/3c27bbd7-b305-4ab0-8a85-5061363cf632___FREC_Scab 3183_final_masked.jpg]

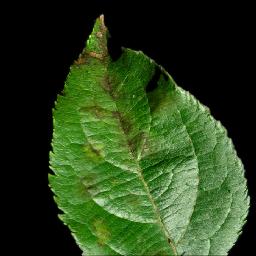

Supplement: Supplemental Information 2 [file peerj-cs-11-2543-s002.zip › Apple/Apple___Apple_scab/3c2f46e3-9f96-4912-a9d5-7a046aa6a1b2___FREC_Scab 2967_final_masked.jpg]

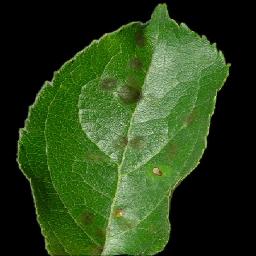

Supplement: Supplemental Information 2 [file peerj-cs-11-2543-s002.zip › Apple/Apple___Apple_scab/3d34bd1c-fae9-446a-a629-147b5e8d5d6b___FREC_Scab 3103_final_masked.jpg]

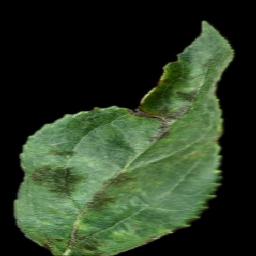

Supplement: Supplemental Information 2 [file peerj-cs-11-2543-s002.zip › Apple/Apple___Apple_scab/3dd3d59c-be2a-4f29-8fa1-d4f37d7fb894___FREC_Scab 3412_final_masked.jpg]

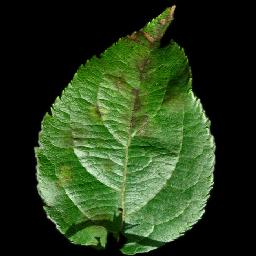

Supplement: Supplemental Information 2 [file peerj-cs-11-2543-s002.zip › Apple/Apple___Apple_scab/3eb26abb-a392-4cc4-9be2-d5e4bb8f3660___FREC_Scab 2969_final_masked.jpg]

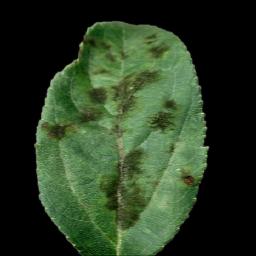

Supplement: Supplemental Information 2 [file peerj-cs-11-2543-s002.zip › Apple/Apple___Apple_scab/3f65aa69-06c9-423f-ac7a-e2ab6edf63ca___FREC_Scab 3360_final_masked.jpg]

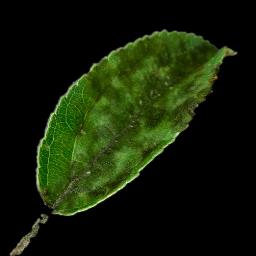

Supplement: Supplemental Information 2 [file peerj-cs-11-2543-s002.zip › Apple/Apple___Apple_scab/4098b029-fcda-4d2f-9703-c16e6e5fae49___FREC_Scab 3455_final_masked.jpg]

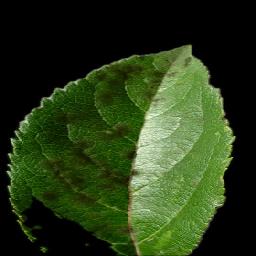

Supplement: Supplemental Information 2 [file peerj-cs-11-2543-s002.zip › Apple/Apple___Apple_scab/40a5e87c-ee32-4d67-9b28-926e94638a1d___FREC_Scab 3251_final_masked.jpg]

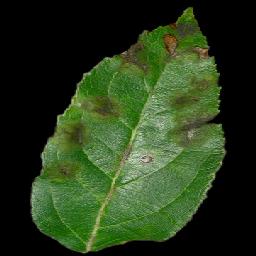

Supplement: Supplemental Information 2 [file peerj-cs-11-2543-s002.zip › Apple/Apple___Apple_scab/40d60cf0-1311-40c7-9e7e-579e74c92027___FREC_Scab 3136_final_masked.jpg]

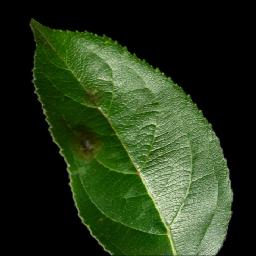

Supplement: Supplemental Information 2 [file peerj-cs-11-2543-s002.zip › Apple/Apple___Apple_scab/40df2c3f-673e-4da8-a172-65fd5a656c1d___FREC_Scab 2957_final_masked.jpg]

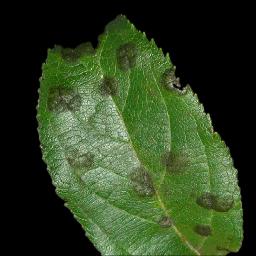

Supplement: Supplemental Information 2 [file peerj-cs-11-2543-s002.zip › Apple/Apple___Apple_scab/4147e694-ce1f-47ea-b641-899e60ef3f3a___FREC_Scab 3278_final_masked.jpg]

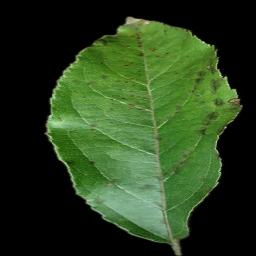

Supplement: Supplemental Information 2 [file peerj-cs-11-2543-s002.zip › Apple/Apple___Apple_scab/4151028c-c8bc-4394-b4a7-aff414864c15___FREC_Scab 3418_final_masked.jpg]

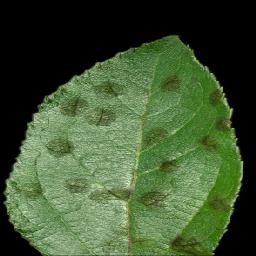

Supplement: Supplemental Information 2 [file peerj-cs-11-2543-s002.zip › Apple/Apple___Apple_scab/419c16b2-13aa-485b-8342-3760707e21f9___FREC_Scab 3285_final_masked.jpg]

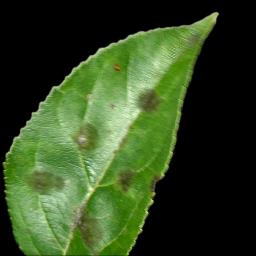

Supplement: Supplemental Information 2 [file peerj-cs-11-2543-s002.zip › Apple/Apple___Apple_scab/41c2bb79-44fb-48f4-864f-eec35e3442f5___FREC_Scab 2931_final_masked.jpg]

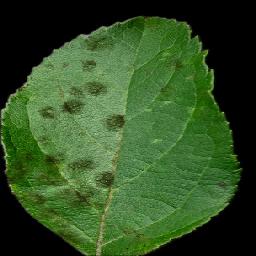

Supplement: Supplemental Information 2 [file peerj-cs-11-2543-s002.zip › Apple/Apple___Apple_scab/41c30a63-9e26-4ced-8110-038a2e36b095___FREC_Scab 3298_final_masked.jpg]

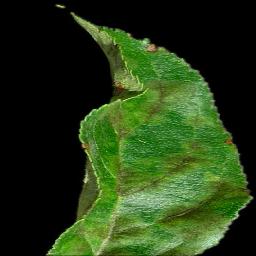

Supplement: Supplemental Information 2 [file peerj-cs-11-2543-s002.zip › Apple/Apple___Apple_scab/43712608-0fa2-4be2-91a0-242aca469cb4___FREC_Scab 2985_final_masked.jpg]

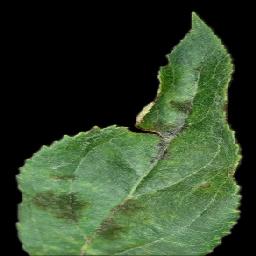

Supplement: Supplemental Information 2 [file peerj-cs-11-2543-s002.zip › Apple/Apple___Apple_scab/43f68711-decd-4831-bf5c-99fa40abe339___FREC_Scab 3413_final_masked.jpg]

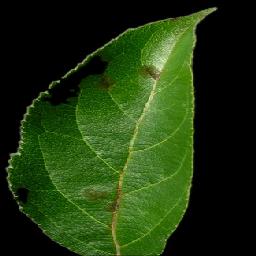

Supplement: Supplemental Information 2 [file peerj-cs-11-2543-s002.zip › Apple/Apple___Apple_scab/4442b558-4db4-4de3-82c3-7524b59bb315___FREC_Scab 2961_final_masked.jpg]

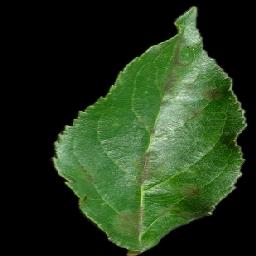

Supplement: Supplemental Information 2 [file peerj-cs-11-2543-s002.zip › Apple/Apple___Apple_scab/444c4ed0-3bee-4059-870a-a15a00f0e37e___FREC_Scab 3067_final_masked.jpg]

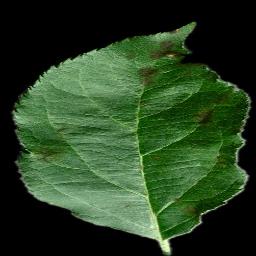

Supplement: Supplemental Information 2 [file peerj-cs-11-2543-s002.zip › Apple/Apple___Apple_scab/4495850d-4478-4680-a657-c082a8ea69ed___FREC_Scab 3534_final_masked.jpg]

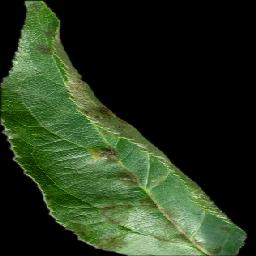

Supplement: Supplemental Information 2 [file peerj-cs-11-2543-s002.zip › Apple/Apple___Apple_scab/44b52928-c5dd-48f4-b2c8-b128032c49dd___FREC_Scab 3518_final_masked.jpg]

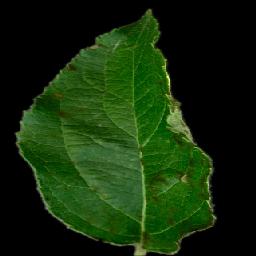

Supplement: Supplemental Information 2 [file peerj-cs-11-2543-s002.zip › Apple/Apple___Apple_scab/44d28651-473c-4e9b-81ec-212893669fba___FREC_Scab 3477_final_masked.jpg]

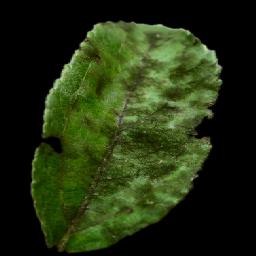

Supplement: Supplemental Information 2 [file peerj-cs-11-2543-s002.zip › Apple/Apple___Apple_scab/44dbb7a4-4c41-4d4c-9e34-9e665a35926c___FREC_Scab 3438_final_masked.jpg]

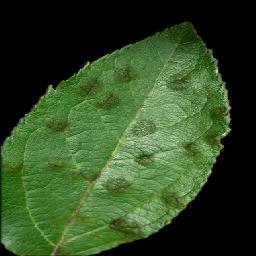

Supplement: Supplemental Information 2 [file peerj-cs-11-2543-s002.zip › Apple/Apple___Apple_scab/44dcf367-d162-407e-8ea8-e363ab8ff5c4___FREC_Scab 3282_final_masked.jpg]

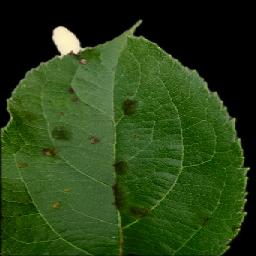

Supplement: Supplemental Information 2 [file peerj-cs-11-2543-s002.zip › Apple/Apple___Apple_scab/45000608-507a-46f5-be52-3ee24d0fb47d___FREC_Scab 3113_final_masked.jpg]

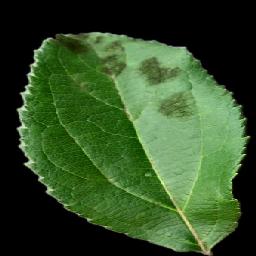

Supplement: Supplemental Information 2 [file peerj-cs-11-2543-s002.zip › Apple/Apple___Apple_scab/4512edee-9d3b-451e-845d-86666feebe99___FREC_Scab 3328_final_masked.jpg]

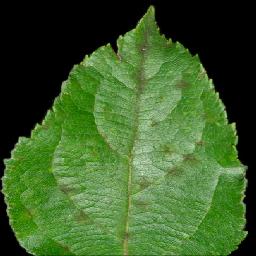

Supplement: Supplemental Information 2 [file peerj-cs-11-2543-s002.zip › Apple/Apple___Apple_scab/453b09e4-6469-47f9-8599-f87e1d6eddf3___FREC_Scab 3048_final_masked.jpg]

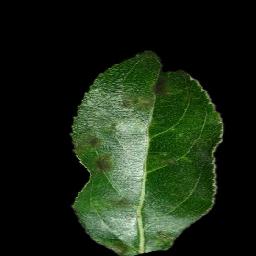

Supplement: Supplemental Information 2 [file peerj-cs-11-2543-s002.zip › Apple/Apple___Apple_scab/45512c84-e453-459c-b12f-1d08ce44001b___FREC_Scab 3072_final_masked.jpg]

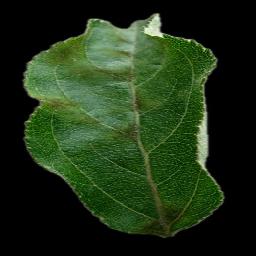

Supplement: Supplemental Information 2 [file peerj-cs-11-2543-s002.zip › Apple/Apple___Apple_scab/4555c009-6c33-4913-accc-fd1a8355646b___FREC_Scab 2929_final_masked.jpg]

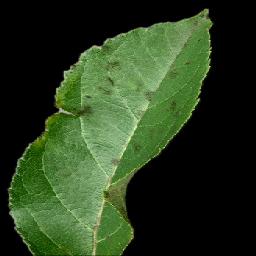

Supplement: Supplemental Information 2 [file peerj-cs-11-2543-s002.zip › Apple/Apple___Apple_scab/45a699f2-a02d-4f1f-b628-6d445c58a595___FREC_Scab 3291_final_masked.jpg]

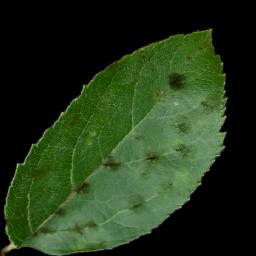

Supplement: Supplemental Information 2 [file peerj-cs-11-2543-s002.zip › Apple/Apple___Apple_scab/45e34a1f-1d46-42e8-921d-c82f2c9e7cd1___FREC_Scab 3228_final_masked.jpg]

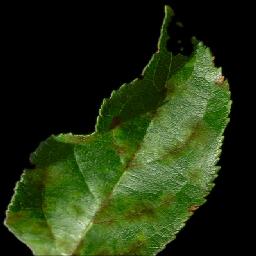

Supplement: Supplemental Information 2 [file peerj-cs-11-2543-s002.zip › Apple/Apple___Apple_scab/45ee8aaa-1a08-46ed-8ac8-bba8e40c61d6___FREC_Scab 2986_final_masked.jpg]

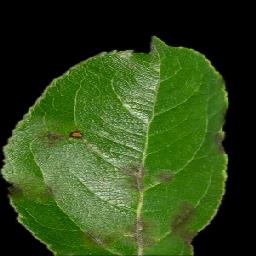

Supplement: Supplemental Information 2 [file peerj-cs-11-2543-s002.zip › Apple/Apple___Apple_scab/461eeeb9-5bf1-4c0a-a7dd-6967917b4537___FREC_Scab 3098_final_masked.jpg]

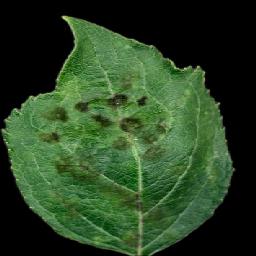

Supplement: Supplemental Information 2 [file peerj-cs-11-2543-s002.zip › Apple/Apple___Apple_scab/462f0ef8-5460-4f5c-8935-62a204d3fc08___FREC_Scab 2924_final_masked.jpg]

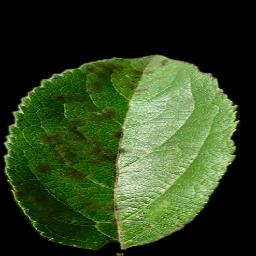

Supplement: Supplemental Information 2 [file peerj-cs-11-2543-s002.zip › Apple/Apple___Apple_scab/470bece4-7eb2-4ec1-917a-278559afe463___FREC_Scab 3250_final_masked.jpg]

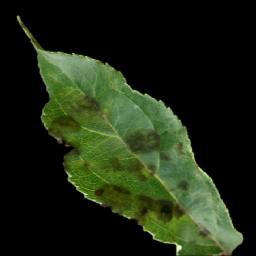

Supplement: Supplemental Information 2 [file peerj-cs-11-2543-s002.zip › Apple/Apple___Apple_scab/474cf33e-0289-4014-bb8a-8004b87b471b___FREC_Scab 3425_final_masked.jpg]

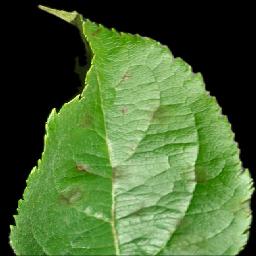

Supplement: Supplemental Information 2 [file peerj-cs-11-2543-s002.zip › Apple/Apple___Apple_scab/47b3d8bb-2461-4593-a0fe-a6adf0925a9a___FREC_Scab 2974_final_masked.jpg]

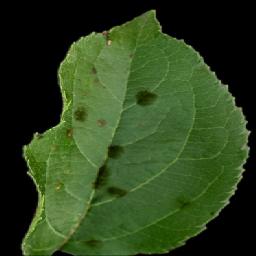

Supplement: Supplemental Information 2 [file peerj-cs-11-2543-s002.zip › Apple/Apple___Apple_scab/47ebb7a4-a8e0-4a57-95e5-dd8e9d1235a2___FREC_Scab 3147_final_masked.jpg]

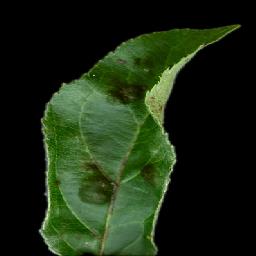

Supplement: Supplemental Information 2 [file peerj-cs-11-2543-s002.zip › Apple/Apple___Apple_scab/48420763-bd18-49b4-9ab4-69319b5733f2___FREC_Scab 3201_final_masked.jpg]

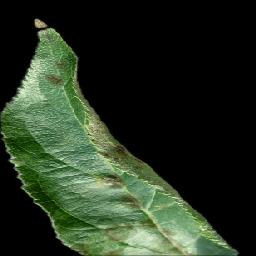

Supplement: Supplemental Information 2 [file peerj-cs-11-2543-s002.zip › Apple/Apple___Apple_scab/48dcb319-8b65-4bac-bba4-aa8ffa8abdd0___FREC_Scab 3519_final_masked.jpg]

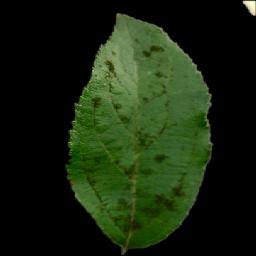

Supplement: Supplemental Information 2 [file peerj-cs-11-2543-s002.zip › Apple/Apple___Apple_scab/48e62e54-d02a-41bb-8486-234408706e69___FREC_Scab 3190_final_masked.jpg]

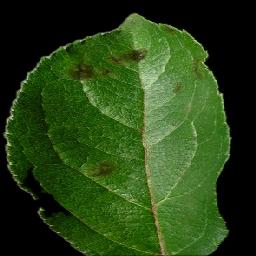

Supplement: Supplemental Information 2 [file peerj-cs-11-2543-s002.zip › Apple/Apple___Apple_scab/4940cd87-695e-4f4d-8a45-5490812447cc___FREC_Scab 3044_final_masked.jpg]

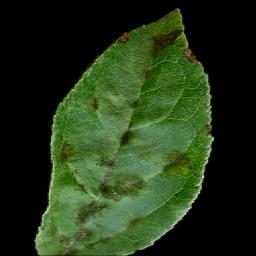

Supplement: Supplemental Information 2 [file peerj-cs-11-2543-s002.zip › Apple/Apple___Apple_scab/495816a8-4d85-47a5-9aca-a09f3f30a068___FREC_Scab 3443_final_masked.jpg]

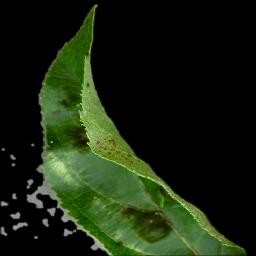

Supplement: Supplemental Information 2 [file peerj-cs-11-2543-s002.zip › Apple/Apple___Apple_scab/4a50158d-dcd0-4a1f-885e-2d9b16a4e344___FREC_Scab 3205_final_masked.jpg]

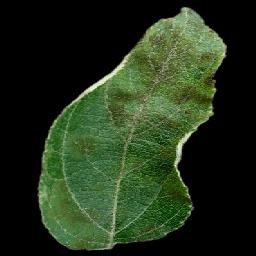

Supplement: Supplemental Information 2 [file peerj-cs-11-2543-s002.zip › Apple/Apple___Apple_scab/4a7f12ff-a785-4d12-8943-f1644789a21b___FREC_Scab 2916_final_masked.jpg]

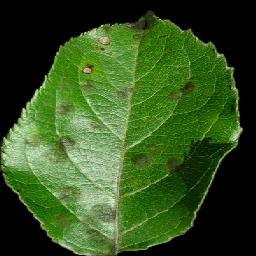

Supplement: Supplemental Information 2 [file peerj-cs-11-2543-s002.zip › Apple/Apple___Apple_scab/4abca5eb-2376-4551-be42-25bbb5400fc7___FREC_Scab 3268_final_masked.jpg]

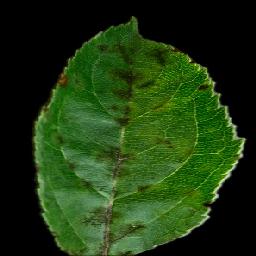

Supplement: Supplemental Information 2 [file peerj-cs-11-2543-s002.zip › Apple/Apple___Apple_scab/4b4b59d5-3c6d-4ce5-b349-616fecb850ff___FREC_Scab 3447_final_masked.jpg]

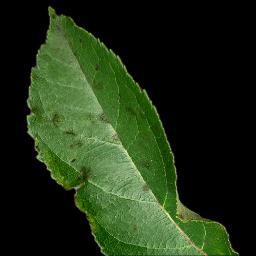

Supplement: Supplemental Information 2 [file peerj-cs-11-2543-s002.zip › Apple/Apple___Apple_scab/4b51cc5d-4122-451b-8598-81ea29dc7cd4___FREC_Scab 3290_final_masked.jpg]

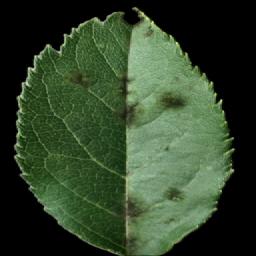

Supplement: Supplemental Information 2 [file peerj-cs-11-2543-s002.zip › Apple/Apple___Apple_scab/4b7c40cd-b145-4945-b649-3576a1d898dc___FREC_Scab 3343_final_masked.jpg]

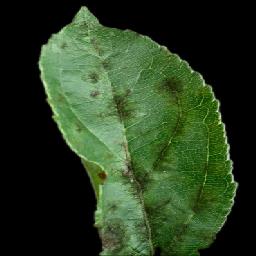

Supplement: Supplemental Information 2 [file peerj-cs-11-2543-s002.zip › Apple/Apple___Apple_scab/4bb38e05-1d5a-40c6-8638-10db134db682___FREC_Scab 3390_final_masked.jpg]

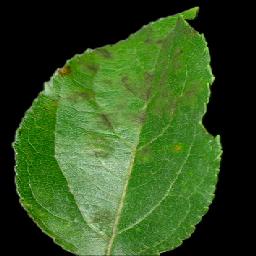

Supplement: Supplemental Information 2 [file peerj-cs-11-2543-s002.zip › Apple/Apple___Apple_scab/4be9d1d9-2170-4856-a81e-e53e7aa5dbf7___FREC_Scab 3082_final_masked.jpg]

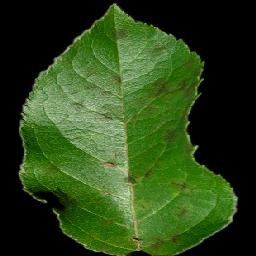

Supplement: Supplemental Information 2 [file peerj-cs-11-2543-s002.zip › Apple/Apple___Apple_scab/4d02c4a9-4b0d-4b4b-9695-91f08228ee53___FREC_Scab 3274_final_masked.jpg]

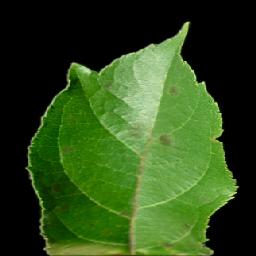

Supplement: Supplemental Information 2 [file peerj-cs-11-2543-s002.zip › Apple/Apple___Apple_scab/4d3bcf15-cc49-4189-82e1-780efdf625a8___FREC_Scab 3254_final_masked.jpg]

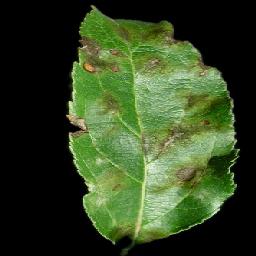

Supplement: Supplemental Information 2 [file peerj-cs-11-2543-s002.zip › Apple/Apple___Apple_scab/4e2c27a7-4be1-4c86-a07b-bc2a64c1bff9___FREC_Scab 3018_final_masked.jpg]

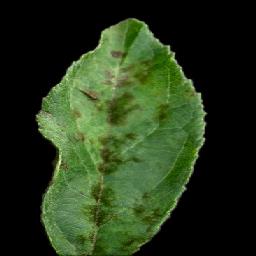

Supplement: Supplemental Information 2 [file peerj-cs-11-2543-s002.zip › Apple/Apple___Apple_scab/4e770559-e332-470e-aae3-c7ad0399dd20___FREC_Scab 3513_final_masked.jpg]

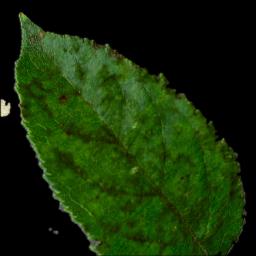

Supplement: Supplemental Information 2 [file peerj-cs-11-2543-s002.zip › Apple/Apple___Apple_scab/50dca3b7-fe83-426b-9fa7-a4e80df022aa___FREC_Scab 3488_final_masked.jpg]

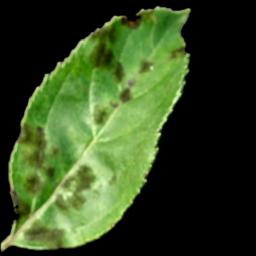

Supplement: Supplemental Information 2 [file peerj-cs-11-2543-s002.zip › Apple/Apple___Apple_scab/51530f6a-2bee-4008-8e77-eeda281e9195___FREC_Scab 3209_final_masked.jpg]

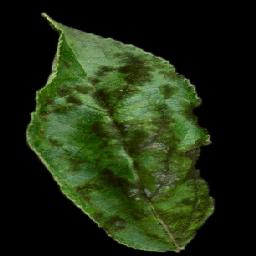

Supplement: Supplemental Information 2 [file peerj-cs-11-2543-s002.zip › Apple/Apple___Apple_scab/517d0958-b10e-4260-a736-7a4bbb9706c0___FREC_Scab 3497_final_masked.jpg]

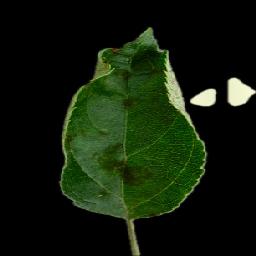

Supplement: Supplemental Information 2 [file peerj-cs-11-2543-s002.zip › Apple/Apple___Apple_scab/518ab601-3736-4d3b-9770-a0efba601c0e___FREC_Scab 3231_final_masked.jpg]

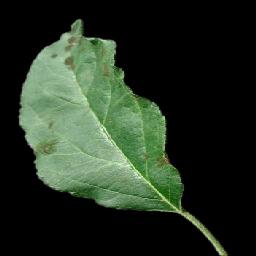

Supplement: Supplemental Information 2 [file peerj-cs-11-2543-s002.zip › Apple/Apple___Apple_scab/51929389-451f-4c78-a66b-b644e82a33b6___FREC_Scab 3528_final_masked.jpg]

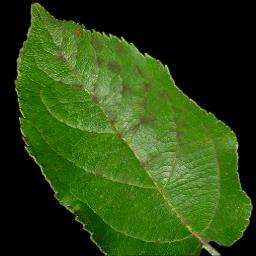

Supplement: Supplemental Information 2 [file peerj-cs-11-2543-s002.zip › Apple/Apple___Apple_scab/52d5723a-b498-4759-89a1-a7d815689716___FREC_Scab 3034_final_masked.jpg]

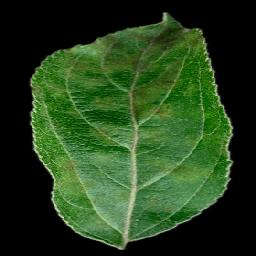

Supplement: Supplemental Information 2 [file peerj-cs-11-2543-s002.zip › Apple/Apple___Apple_scab/53035d10-9f2b-4c90-ab75-dfde2a9af82f___FREC_Scab 2915_final_masked.jpg]

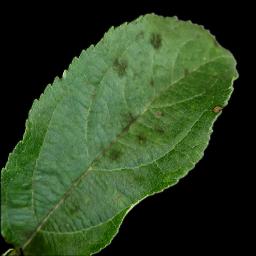

Supplement: Supplemental Information 2 [file peerj-cs-11-2543-s002.zip › Apple/Apple___Apple_scab/5345e6fb-1829-457f-ac86-6683d762c9ea___FREC_Scab 3482_final_masked.jpg]

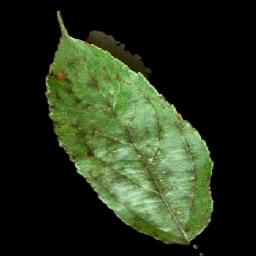

Supplement: Supplemental Information 2 [file peerj-cs-11-2543-s002.zip › Apple/Apple___Apple_scab/536ce9e9-6c98-42c6-9447-f38073b3e9da___FREC_Scab 3222_final_masked.jpg]

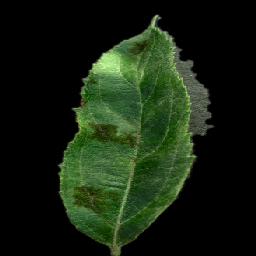

Supplement: Supplemental Information 2 [file peerj-cs-11-2543-s002.zip › Apple/Apple___Apple_scab/537873ed-fc63-4df3-bb6a-a5bf4c742619___FREC_Scab 3505_final_masked.jpg]

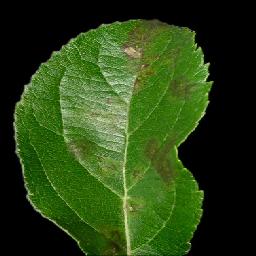

Supplement: Supplemental Information 2 [file peerj-cs-11-2543-s002.zip › Apple/Apple___Apple_scab/53c67c39-c05f-4b2e-939e-23d44a32efac___FREC_Scab 3087_final_masked.jpg]

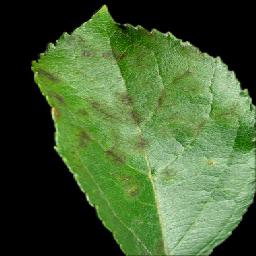

Supplement: Supplemental Information 2 [file peerj-cs-11-2543-s002.zip › Apple/Apple___Apple_scab/53d37faf-7a68-4fe2-ac1f-5438637742d4___FREC_Scab 3080_final_masked.jpg]

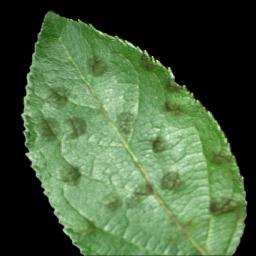

Supplement: Supplemental Information 2 [file peerj-cs-11-2543-s002.zip › Apple/Apple___Apple_scab/53fe6519-da0a-4183-80d6-a18828a33223___FREC_Scab 3283_final_masked.jpg]

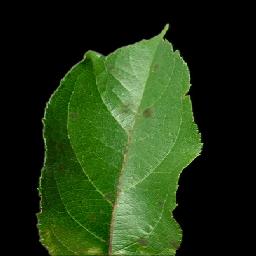

Supplement: Supplemental Information 2 [file peerj-cs-11-2543-s002.zip › Apple/Apple___Apple_scab/550b0aab-e777-4942-a186-fc910e0fa89b___FREC_Scab 3253_final_masked.jpg]
