# Supplementary figures and images for: Iterative segmentation and classification for enhanced crop disease diagnosis using optimized hybrid U-Nets model (part 1 of 2)
Source: PeerJ Comput Sci. 2025 Jun 11;11:e2543. doi: 10.7717/peerj-cs.2543 (PMC12190645; doi:10.7717/peerj-cs.2543)

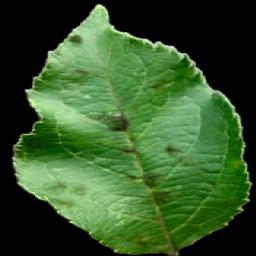

Supplement: Supplemental Information 2 [file peerj-cs-11-2543-s002.zip › Apple/Apple___Apple_scab/00075aa8-d81a-4184-8541-b692b78d398a___FREC_Scab 3335_final_masked.jpg]

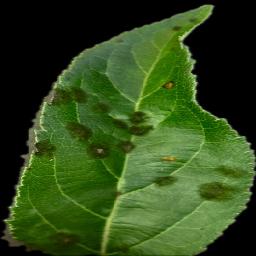

Supplement: Supplemental Information 2 [file peerj-cs-11-2543-s002.zip › Apple/Apple___Apple_scab/01a66316-0e98-4d3b-a56f-d78752cd043f___FREC_Scab 3003_final_masked.jpg]

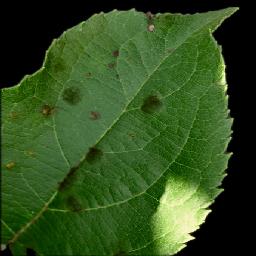

Supplement: Supplemental Information 2 [file peerj-cs-11-2543-s002.zip › Apple/Apple___Apple_scab/01f3deaa-6143-4b6c-9c22-620a46d8be04___FREC_Scab 3112_final_masked.jpg]

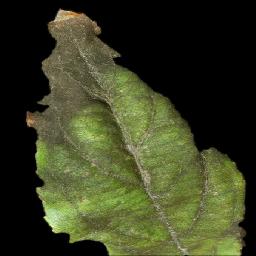

Supplement: Supplemental Information 2 [file peerj-cs-11-2543-s002.zip › Apple/Apple___Apple_scab/0208f4eb-45a4-4399-904e-989ac2c6257c___FREC_Scab 3037_final_masked.jpg]

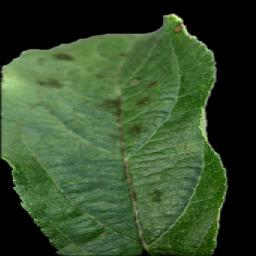

Supplement: Supplemental Information 2 [file peerj-cs-11-2543-s002.zip › Apple/Apple___Apple_scab/023123cb-7b69-4c9f-a521-766d7c8543bb___FREC_Scab 3487_final_masked.jpg]

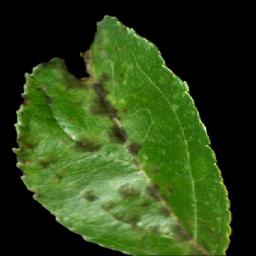

Supplement: Supplemental Information 2 [file peerj-cs-11-2543-s002.zip › Apple/Apple___Apple_scab/0261a6e4-21f8-481a-8827-b674e6955644___FREC_Scab 3055_final_masked.jpg]

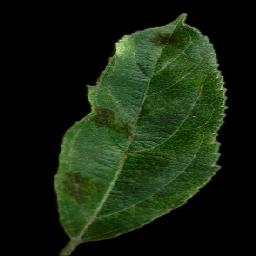

Supplement: Supplemental Information 2 [file peerj-cs-11-2543-s002.zip › Apple/Apple___Apple_scab/029424b0-0ef5-491b-9ef5-069190d24d8f___FREC_Scab 3504_final_masked.jpg]

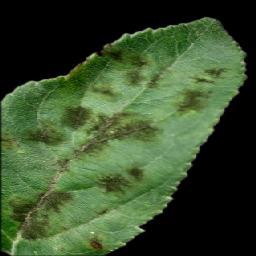

Supplement: Supplemental Information 2 [file peerj-cs-11-2543-s002.zip › Apple/Apple___Apple_scab/03354abb-aa1c-4f9d-a1ef-9f40505cd539___FREC_Scab 3355_final_masked.jpg]

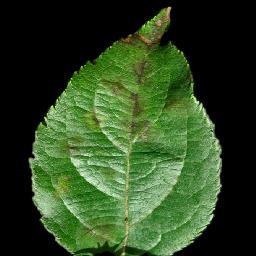

Supplement: Supplemental Information 2 [file peerj-cs-11-2543-s002.zip › Apple/Apple___Apple_scab/0340dc35-5215-48ab-8db7-06af99fcb358___FREC_Scab 2966_final_masked.jpg]

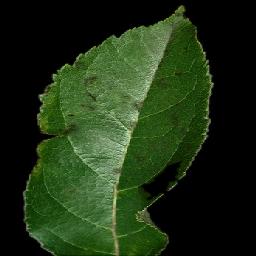

Supplement: Supplemental Information 2 [file peerj-cs-11-2543-s002.zip › Apple/Apple___Apple_scab/0395b847-2c73-4674-826f-33a6afb5b4fe___FREC_Scab 3287_final_masked.jpg]

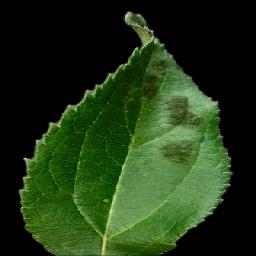

Supplement: Supplemental Information 2 [file peerj-cs-11-2543-s002.zip › Apple/Apple___Apple_scab/03eccb1a-0368-4ac7-9f48-7546037b775a___FREC_Scab 3334_final_masked.jpg]

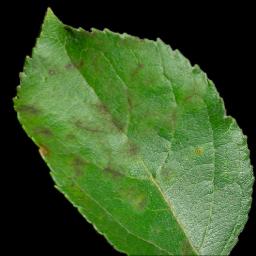

Supplement: Supplemental Information 2 [file peerj-cs-11-2543-s002.zip › Apple/Apple___Apple_scab/0537ad56-3d24-4e97-a947-65b9b37f8988___FREC_Scab 3079_final_masked.jpg]

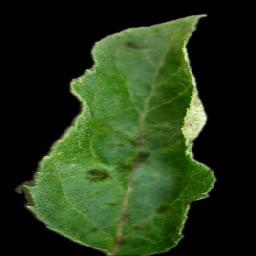

Supplement: Supplemental Information 2 [file peerj-cs-11-2543-s002.zip › Apple/Apple___Apple_scab/058d5e64-2c57-45ba-94cb-ac83fd1885a0___FREC_Scab 3181_final_masked.jpg]

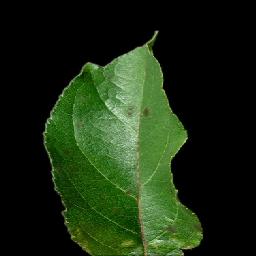

Supplement: Supplemental Information 2 [file peerj-cs-11-2543-s002.zip › Apple/Apple___Apple_scab/0631708e-5bac-4611-8ff9-6d5ee87ce3b3___FREC_Scab 3252_final_masked.jpg]

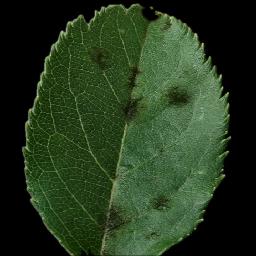

Supplement: Supplemental Information 2 [file peerj-cs-11-2543-s002.zip › Apple/Apple___Apple_scab/0672ab32-9fce-41f3-ae69-e39c48a0a292___FREC_Scab 3347_final_masked.jpg]

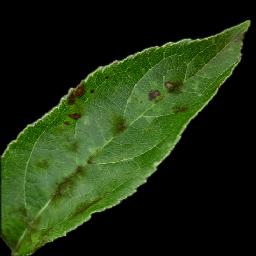

Supplement: Supplemental Information 2 [file peerj-cs-11-2543-s002.zip › Apple/Apple___Apple_scab/06cf74b6-d895-4a09-b3b1-bc63579f8c33___FREC_Scab 3128_final_masked.jpg]

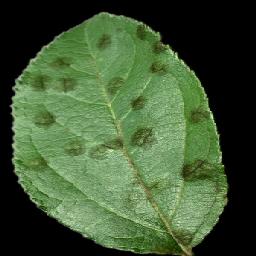

Supplement: Supplemental Information 2 [file peerj-cs-11-2543-s002.zip › Apple/Apple___Apple_scab/073b509f-c1ea-4e61-881b-2e874c5f4199___FREC_Scab 3286_final_masked.jpg]

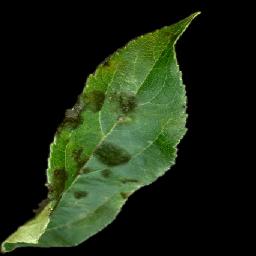

Supplement: Supplemental Information 2 [file peerj-cs-11-2543-s002.zip › Apple/Apple___Apple_scab/075b1885-250b-4b3e-8168-e54efdbb04e8___FREC_Scab 3173_final_masked.jpg]

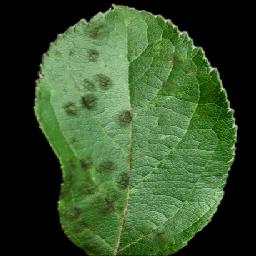

Supplement: Supplemental Information 2 [file peerj-cs-11-2543-s002.zip › Apple/Apple___Apple_scab/0812e1e4-6d1b-4264-8f23-6519354fc5a6___FREC_Scab 3293_final_masked.jpg]

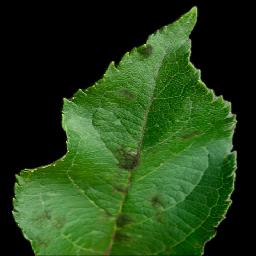

Supplement: Supplemental Information 2 [file peerj-cs-11-2543-s002.zip › Apple/Apple___Apple_scab/0896ecbc-1af2-4868-8279-ed302acb29bc___FREC_Scab 3339_final_masked.jpg]

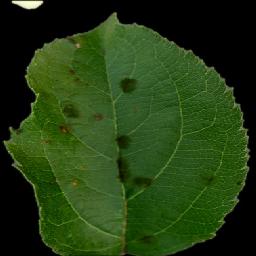

Supplement: Supplemental Information 2 [file peerj-cs-11-2543-s002.zip › Apple/Apple___Apple_scab/08c42d78-aa7b-4106-b0c1-b260f898dcba___FREC_Scab 3151_final_masked.jpg]

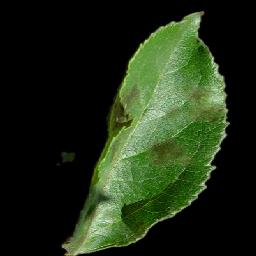

Supplement: Supplemental Information 2 [file peerj-cs-11-2543-s002.zip › Apple/Apple___Apple_scab/095c9131-a0cb-47de-898a-f9371f208c01___FREC_Scab 3109_final_masked.jpg]

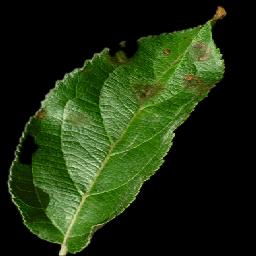

Supplement: Supplemental Information 2 [file peerj-cs-11-2543-s002.zip › Apple/Apple___Apple_scab/09ba6ad9-aa8d-4493-b1bf-818a4b30c4aa___FREC_Scab 2981_final_masked.jpg]

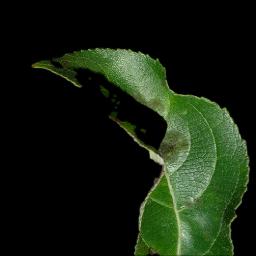

Supplement: Supplemental Information 2 [file peerj-cs-11-2543-s002.zip › Apple/Apple___Apple_scab/09e40e4f-f8bc-4537-b20d-0b244760dbf4___FREC_Scab 3216_final_masked.jpg]

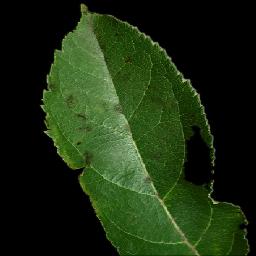

Supplement: Supplemental Information 2 [file peerj-cs-11-2543-s002.zip › Apple/Apple___Apple_scab/0a14783a-838a-4d4f-a671-ff98011714c6___FREC_Scab 3288_final_masked.jpg]

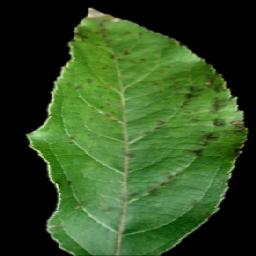

Supplement: Supplemental Information 2 [file peerj-cs-11-2543-s002.zip › Apple/Apple___Apple_scab/0a5e9323-dbad-432d-ac58-d291718345d9___FREC_Scab 3417_final_masked.jpg]

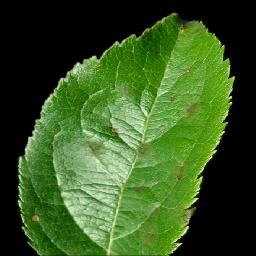

Supplement: Supplemental Information 2 [file peerj-cs-11-2543-s002.zip › Apple/Apple___Apple_scab/0a6812de-7416-4ffe-aba9-307599a02c84___FREC_Scab 2973_final_masked.jpg]

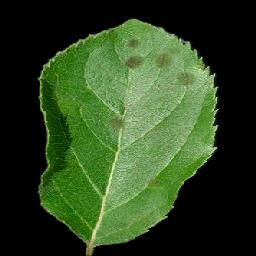

Supplement: Supplemental Information 2 [file peerj-cs-11-2543-s002.zip › Apple/Apple___Apple_scab/0a769a71-052a-4f19-a4d8-b0f0cb75541c___FREC_Scab 3165_final_masked.jpg]

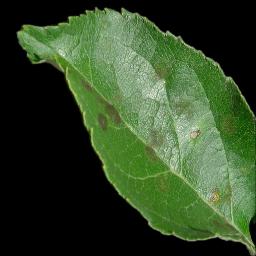

Supplement: Supplemental Information 2 [file peerj-cs-11-2543-s002.zip › Apple/Apple___Apple_scab/0b170906-9436-4c0d-84c1-c396ad9d909b___FREC_Scab 3101_final_masked.jpg]

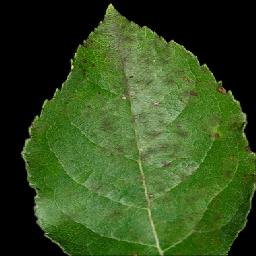

Supplement: Supplemental Information 2 [file peerj-cs-11-2543-s002.zip › Apple/Apple___Apple_scab/0b1e31fa-cbc0-41ed-9139-c794e6855e82___FREC_Scab 3089_final_masked.jpg]

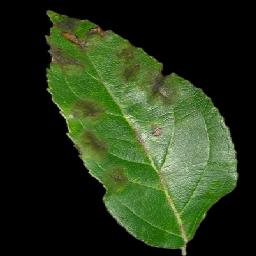

Supplement: Supplemental Information 2 [file peerj-cs-11-2543-s002.zip › Apple/Apple___Apple_scab/0b4a52e3-e15e-4117-b2e8-7cdb5dca3ce9___FREC_Scab 3137_final_masked.jpg]

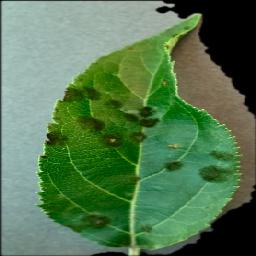

Supplement: Supplemental Information 2 [file peerj-cs-11-2543-s002.zip › Apple/Apple___Apple_scab/0c620ec5-11cf-4120-94ab-1311e99df147___FREC_Scab 3131_final_masked.jpg]

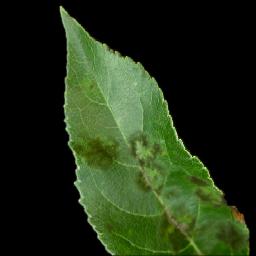

Supplement: Supplemental Information 2 [file peerj-cs-11-2543-s002.zip › Apple/Apple___Apple_scab/0cbfa4fa-63d8-43ce-9385-ff140e524b69___FREC_Scab 3164_final_masked.jpg]

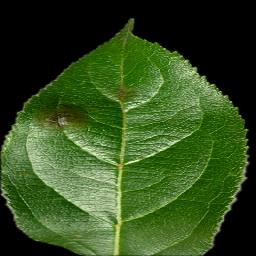

Supplement: Supplemental Information 2 [file peerj-cs-11-2543-s002.zip › Apple/Apple___Apple_scab/0d3c0790-7833-470b-ac6e-94d0a3bf3e7c___FREC_Scab 2959_final_masked.jpg]

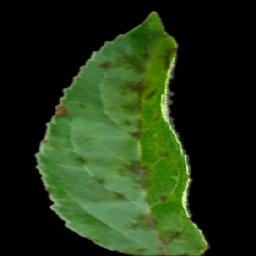

Supplement: Supplemental Information 2 [file peerj-cs-11-2543-s002.zip › Apple/Apple___Apple_scab/0d8d5b80-962d-4381-8d3b-9eca3f2f1bb0___FREC_Scab 3449_final_masked.jpg]

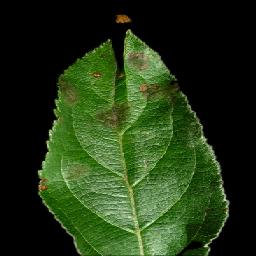

Supplement: Supplemental Information 2 [file peerj-cs-11-2543-s002.zip › Apple/Apple___Apple_scab/0db71c1d-93d7-4481-b0d0-b73f995131a9___FREC_Scab 2976_final_masked.jpg]

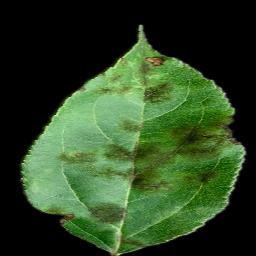

Supplement: Supplemental Information 2 [file peerj-cs-11-2543-s002.zip › Apple/Apple___Apple_scab/0e90fe4a-e8b6-4186-9429-a9fea180af9a___FREC_Scab 3391_final_masked.jpg]

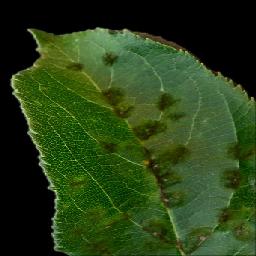

Supplement: Supplemental Information 2 [file peerj-cs-11-2543-s002.zip › Apple/Apple___Apple_scab/0ea78733-9404-4536-8793-a108c66269b3___FREC_Scab 3145_final_masked.jpg]

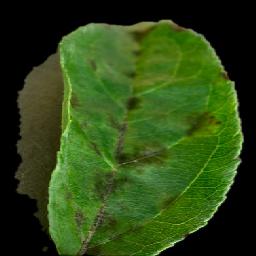

Supplement: Supplemental Information 2 [file peerj-cs-11-2543-s002.zip › Apple/Apple___Apple_scab/10072248-60e2-4190-b4df-14e8ab61008c___FREC_Scab 3445_final_masked.jpg]

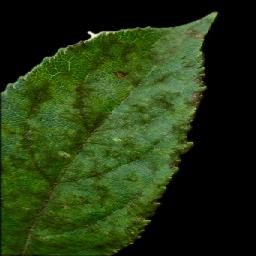

Supplement: Supplemental Information 2 [file peerj-cs-11-2543-s002.zip › Apple/Apple___Apple_scab/106aaa5f-c98d-482d-88ef-8e5170e9072d___FREC_Scab 3489_final_masked.jpg]

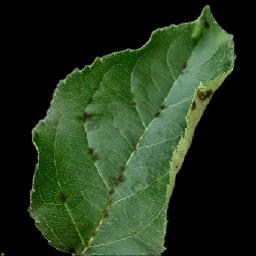

Supplement: Supplemental Information 2 [file peerj-cs-11-2543-s002.zip › Apple/Apple___Apple_scab/11c50257-617e-4a6a-a996-2b195af9ada4___FREC_Scab 3462_final_masked.jpg]

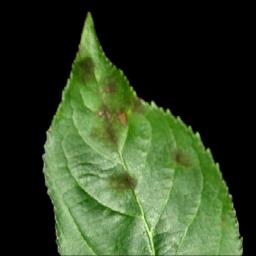

Supplement: Supplemental Information 2 [file peerj-cs-11-2543-s002.zip › Apple/Apple___Apple_scab/11f5b020-3dfd-400c-9819-7d229bc5f929___FREC_Scab 2945_final_masked.jpg]

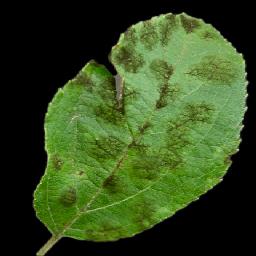

Supplement: Supplemental Information 2 [file peerj-cs-11-2543-s002.zip › Apple/Apple___Apple_scab/12bc3d5c-2409-426e-b6cb-a08cbf488153___FREC_Scab 3154_final_masked.jpg]

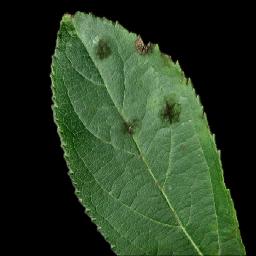

Supplement: Supplemental Information 2 [file peerj-cs-11-2543-s002.zip › Apple/Apple___Apple_scab/133c27cd-57f6-4790-b446-972327e13194___FREC_Scab 3362_final_masked.jpg]

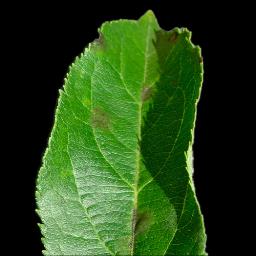

Supplement: Supplemental Information 2 [file peerj-cs-11-2543-s002.zip › Apple/Apple___Apple_scab/13b10b04-7da6-4d34-8b43-258239a25ffd___FREC_Scab 2990_final_masked.jpg]

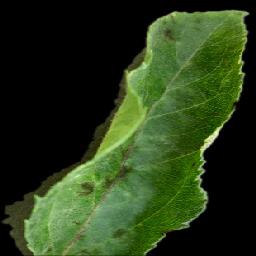

Supplement: Supplemental Information 2 [file peerj-cs-11-2543-s002.zip › Apple/Apple___Apple_scab/13b16e68-c82c-4de8-ab34-877ff352e821___FREC_Scab 3180_final_masked.jpg]

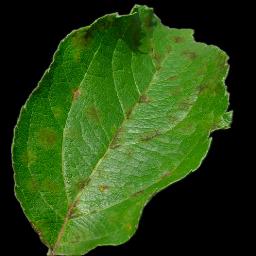

Supplement: Supplemental Information 2 [file peerj-cs-11-2543-s002.zip › Apple/Apple___Apple_scab/13f31401-f65a-4025-823a-4f2738e8bff9___FREC_Scab 3062_final_masked.jpg]

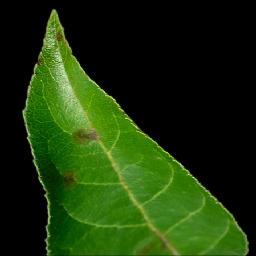

Supplement: Supplemental Information 2 [file peerj-cs-11-2543-s002.zip › Apple/Apple___Apple_scab/14c623e5-051c-42f6-9e4f-f7a93e6a723c___FREC_Scab 2965_final_masked.jpg]

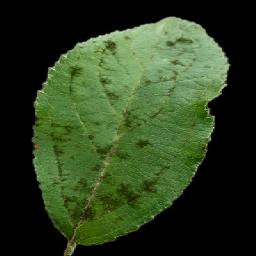

Supplement: Supplemental Information 2 [file peerj-cs-11-2543-s002.zip › Apple/Apple___Apple_scab/1547f817-6a4d-4cbc-8ce0-7dff293562ee___FREC_Scab 3194_final_masked.jpg]

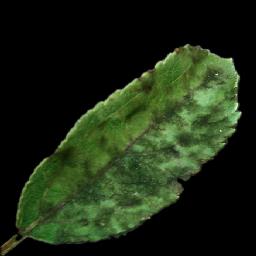

Supplement: Supplemental Information 2 [file peerj-cs-11-2543-s002.zip › Apple/Apple___Apple_scab/154842bb-6ee3-4176-9007-cf7a43cfe140___FREC_Scab 3434_final_masked.jpg]

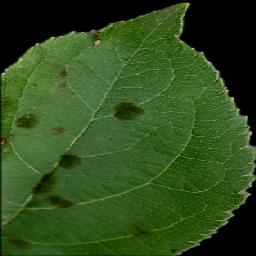

Supplement: Supplemental Information 2 [file peerj-cs-11-2543-s002.zip › Apple/Apple___Apple_scab/15bfa086-7d4a-43c0-800d-2fe8255ba50c___FREC_Scab 3150_final_masked.jpg]

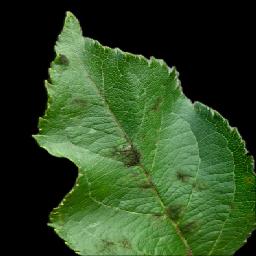

Supplement: Supplemental Information 2 [file peerj-cs-11-2543-s002.zip › Apple/Apple___Apple_scab/162d8ca4-7cd3-46f1-904f-09721df3e498___FREC_Scab 3336_final_masked.jpg]

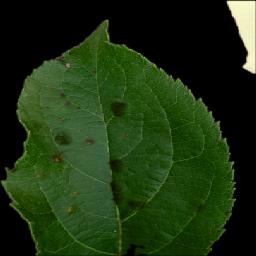

Supplement: Supplemental Information 2 [file peerj-cs-11-2543-s002.zip › Apple/Apple___Apple_scab/1637ad84-640c-41e7-9be7-6d8e7769b875___FREC_Scab 3110_final_masked.jpg]

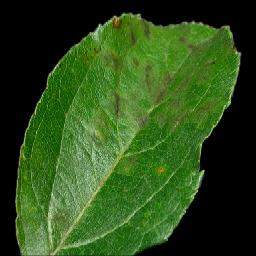

Supplement: Supplemental Information 2 [file peerj-cs-11-2543-s002.zip › Apple/Apple___Apple_scab/176a93ed-6c2d-4828-ada1-87f1688b509b___FREC_Scab 3083_final_masked.jpg]

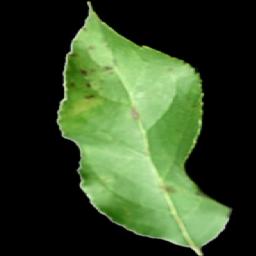

Supplement: Supplemental Information 2 [file peerj-cs-11-2543-s002.zip › Apple/Apple___Apple_scab/18ab74ed-8e95-4787-b2e1-1283594bd00b___FREC_Scab 3012_final_masked.jpg]

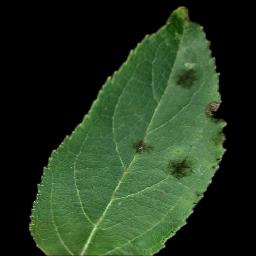

Supplement: Supplemental Information 2 [file peerj-cs-11-2543-s002.zip › Apple/Apple___Apple_scab/18e95262-fb0a-472c-93ab-e03138a360dc___FREC_Scab 3366_final_masked.jpg]

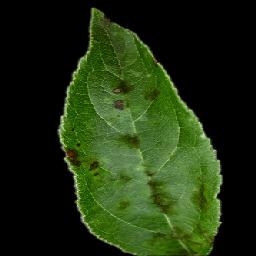

Supplement: Supplemental Information 2 [file peerj-cs-11-2543-s002.zip › Apple/Apple___Apple_scab/18ea82fd-4e16-416f-8ad9-54e0f6601693___FREC_Scab 3127_final_masked.jpg]

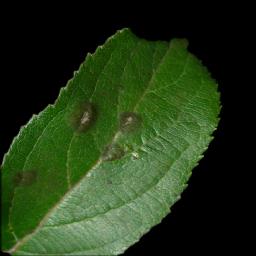

Supplement: Supplemental Information 2 [file peerj-cs-11-2543-s002.zip › Apple/Apple___Apple_scab/1962d1ec-fb66-4583-b631-81cd23ecf063___FREC_Scab 3238_final_masked.jpg]

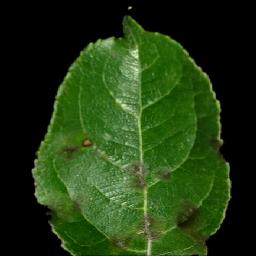

Supplement: Supplemental Information 2 [file peerj-cs-11-2543-s002.zip › Apple/Apple___Apple_scab/1a21aabb-6f74-4644-8d9e-a517568b7e9c___FREC_Scab 3095_final_masked.jpg]

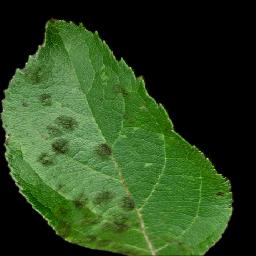

Supplement: Supplemental Information 2 [file peerj-cs-11-2543-s002.zip › Apple/Apple___Apple_scab/1a304331-98b5-473f-bbb1-c33b8441052a___FREC_Scab 3296_final_masked.jpg]

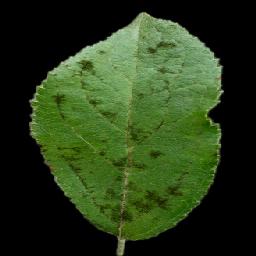

Supplement: Supplemental Information 2 [file peerj-cs-11-2543-s002.zip › Apple/Apple___Apple_scab/1a4047d7-23d6-4bba-ba10-b6e7005ea01b___FREC_Scab 3193_final_masked.jpg]

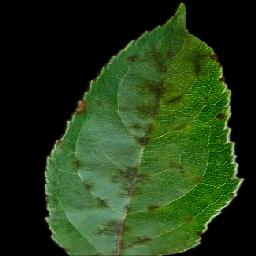

Supplement: Supplemental Information 2 [file peerj-cs-11-2543-s002.zip › Apple/Apple___Apple_scab/1a41bab0-45e0-4dda-a798-9bf4a998f1b6___FREC_Scab 3450_final_masked.jpg]

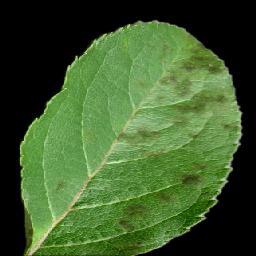

Supplement: Supplemental Information 2 [file peerj-cs-11-2543-s002.zip › Apple/Apple___Apple_scab/1b1004c8-99e9-4c85-8fe5-b1c11d558cf8___FREC_Scab 3300_final_masked.jpg]

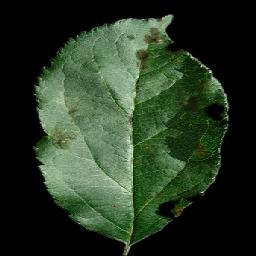

Supplement: Supplemental Information 2 [file peerj-cs-11-2543-s002.zip › Apple/Apple___Apple_scab/1bc60205-27cc-4d10-bc87-7e968573e5d5___FREC_Scab 3531_final_masked.jpg]

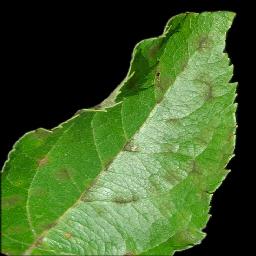

Supplement: Supplemental Information 2 [file peerj-cs-11-2543-s002.zip › Apple/Apple___Apple_scab/1bcc3b5d-7e00-43fb-983e-95ae271a715b___FREC_Scab 3061_final_masked.jpg]

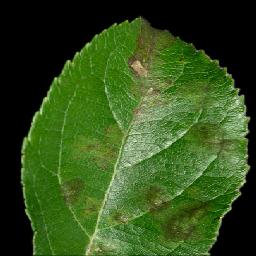

Supplement: Supplemental Information 2 [file peerj-cs-11-2543-s002.zip › Apple/Apple___Apple_scab/1c10ab31-02b9-4008-b66f-9b44d8a9d323___FREC_Scab 3084_final_masked.jpg]

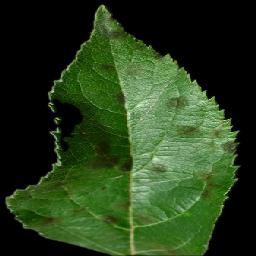

Supplement: Supplemental Information 2 [file peerj-cs-11-2543-s002.zip › Apple/Apple___Apple_scab/1cb869ea-0a4c-47d7-9def-a88c16b72ddc___FREC_Scab 3350_final_masked.jpg]

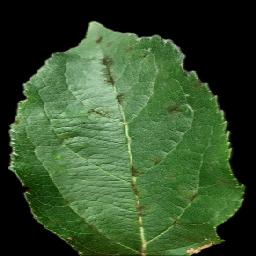

Supplement: Supplemental Information 2 [file peerj-cs-11-2543-s002.zip › Apple/Apple___Apple_scab/1cdf2779-8cb1-4ae4-be70-b0eeb1abb0f8___FREC_Scab 3370_final_masked.jpg]

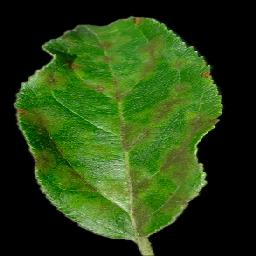

Supplement: Supplemental Information 2 [file peerj-cs-11-2543-s002.zip › Apple/Apple___Apple_scab/1ceeeee0-40bd-4ac9-8a57-59385bf5df8a___FREC_Scab 3001_final_masked.jpg]

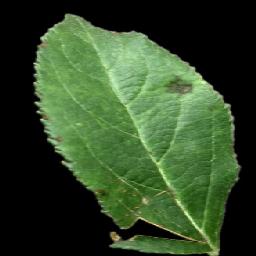

Supplement: Supplemental Information 2 [file peerj-cs-11-2543-s002.zip › Apple/Apple___Apple_scab/1d81b4c1-072d-4cf7-b1ca-ce43f59822d8___FREC_Scab 3406_final_masked.jpg]

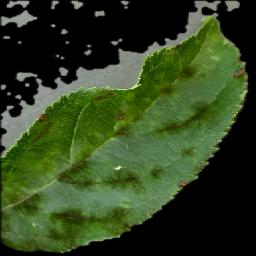

Supplement: Supplemental Information 2 [file peerj-cs-11-2543-s002.zip › Apple/Apple___Apple_scab/1d9d67e2-5603-4710-ae2b-6cb0b922ae61___FREC_Scab 3122_final_masked.jpg]

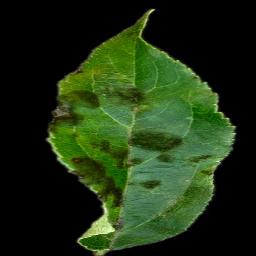

Supplement: Supplemental Information 2 [file peerj-cs-11-2543-s002.zip › Apple/Apple___Apple_scab/1f6abf22-93fa-48f0-a509-cc3e210f75f0___FREC_Scab 3172_final_masked.jpg]

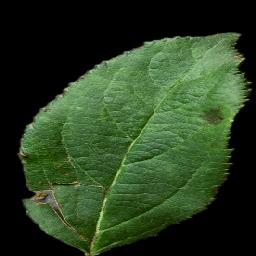

Supplement: Supplemental Information 2 [file peerj-cs-11-2543-s002.zip › Apple/Apple___Apple_scab/1f97d75c-db85-4344-8687-82ca56454d17___FREC_Scab 3408_final_masked.jpg]

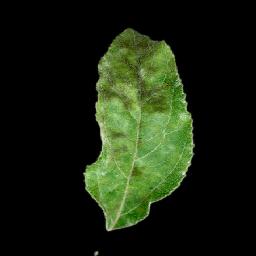

Supplement: Supplemental Information 2 [file peerj-cs-11-2543-s002.zip › Apple/Apple___Apple_scab/1f98b949-4df2-45cf-8572-3b4a753be21a___FREC_Scab 2907_final_masked.jpg]

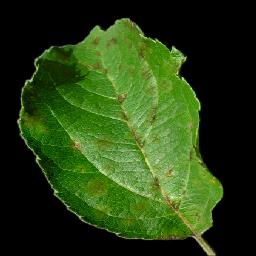

Supplement: Supplemental Information 2 [file peerj-cs-11-2543-s002.zip › Apple/Apple___Apple_scab/201d8b27-5a38-43e5-8065-f7fe039ad781___FREC_Scab 3058_final_masked.jpg]

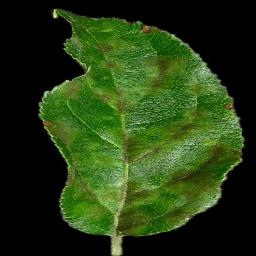

Supplement: Supplemental Information 2 [file peerj-cs-11-2543-s002.zip › Apple/Apple___Apple_scab/20f4f8d1-7527-420b-a90d-e17b9e45549a___FREC_Scab 2997_final_masked.jpg]

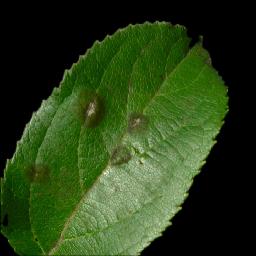

Supplement: Supplemental Information 2 [file peerj-cs-11-2543-s002.zip › Apple/Apple___Apple_scab/222a9802-f14c-433e-9d18-d0668ed3c279___FREC_Scab 3239_final_masked.jpg]

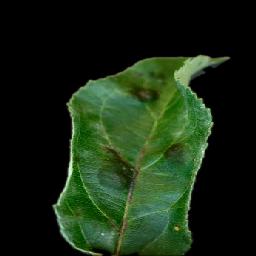

Supplement: Supplemental Information 2 [file peerj-cs-11-2543-s002.zip › Apple/Apple___Apple_scab/22c12d6a-3c17-45be-b53b-f17f1b3f5168___FREC_Scab 3206_final_masked.jpg]

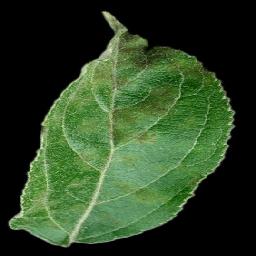

Supplement: Supplemental Information 2 [file peerj-cs-11-2543-s002.zip › Apple/Apple___Apple_scab/233b7aec-ccec-47a3-b5bd-27e154d7a8f7___FREC_Scab 2912_final_masked.jpg]

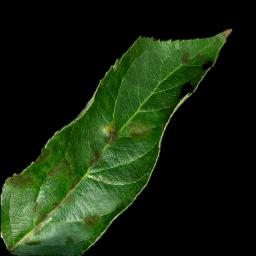

Supplement: Supplemental Information 2 [file peerj-cs-11-2543-s002.zip › Apple/Apple___Apple_scab/23466313-6a2c-46e7-ad89-1f95dd2d3b40___FREC_Scab 3517_final_masked.jpg]

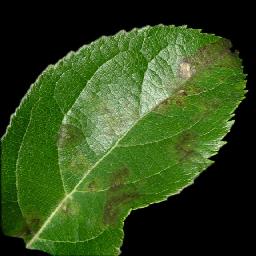

Supplement: Supplemental Information 2 [file peerj-cs-11-2543-s002.zip › Apple/Apple___Apple_scab/23576562-df0c-46bb-8c50-1bf03412097f___FREC_Scab 3086_final_masked.jpg]

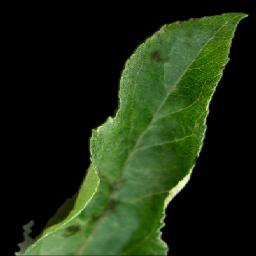

Supplement: Supplemental Information 2 [file peerj-cs-11-2543-s002.zip › Apple/Apple___Apple_scab/23f31e0d-3330-4752-9f83-8aba28c90ccb___FREC_Scab 3178_final_masked.jpg]

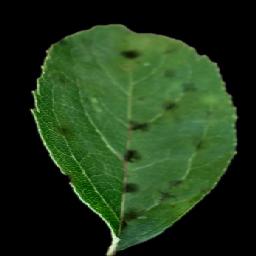

Supplement: Supplemental Information 2 [file peerj-cs-11-2543-s002.zip › Apple/Apple___Apple_scab/2438c1f0-556e-4575-b382-96e17f908e34___FREC_Scab 3230_final_masked.jpg]

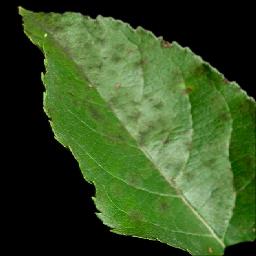

Supplement: Supplemental Information 2 [file peerj-cs-11-2543-s002.zip › Apple/Apple___Apple_scab/246c33b0-fac8-4cca-ae8b-17947338b8b2___FREC_Scab 3091_final_masked.jpg]

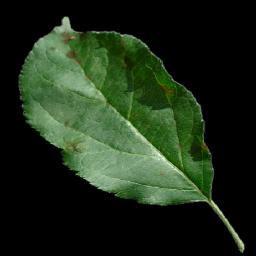

Supplement: Supplemental Information 2 [file peerj-cs-11-2543-s002.zip › Apple/Apple___Apple_scab/24d43ad3-24e6-4236-8ef7-59b268520ca3___FREC_Scab 3533_final_masked.jpg]

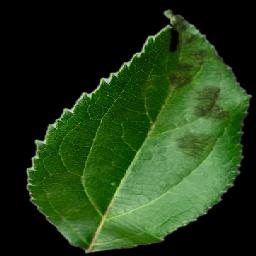

Supplement: Supplemental Information 2 [file peerj-cs-11-2543-s002.zip › Apple/Apple___Apple_scab/252087d7-57c9-4ebe-b6c4-840d6c5dfcb1___FREC_Scab 3329_final_masked.jpg]

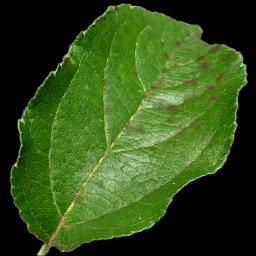

Supplement: Supplemental Information 2 [file peerj-cs-11-2543-s002.zip › Apple/Apple___Apple_scab/258ce9eb-2b67-475b-b09c-0bc83b0987f1___FREC_Scab 3033_final_masked.jpg]

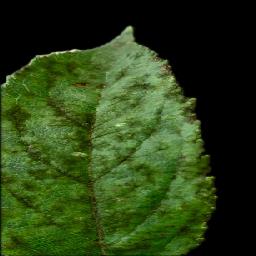

Supplement: Supplemental Information 2 [file peerj-cs-11-2543-s002.zip › Apple/Apple___Apple_scab/25aeb397-bc36-4fe5-affe-6b8195d4f282___FREC_Scab 3494_final_masked.jpg]

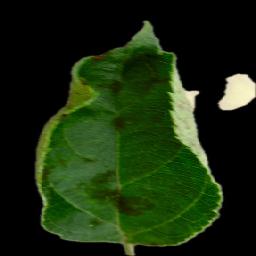

Supplement: Supplemental Information 2 [file peerj-cs-11-2543-s002.zip › Apple/Apple___Apple_scab/268a2ad1-d649-4ec0-add3-61cc8f54f479___FREC_Scab 3235_final_masked.jpg]

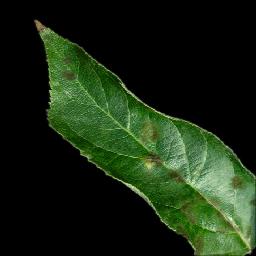

Supplement: Supplemental Information 2 [file peerj-cs-11-2543-s002.zip › Apple/Apple___Apple_scab/26a31865-1d42-4bd6-8b67-02cbedfd6f64___FREC_Scab 3516_final_masked.jpg]

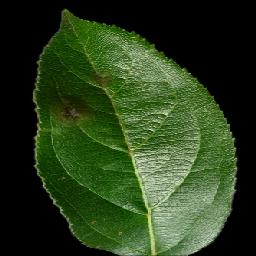

Supplement: Supplemental Information 2 [file peerj-cs-11-2543-s002.zip › Apple/Apple___Apple_scab/270dd6be-95f4-4fd1-8423-b698663b3f72___FREC_Scab 2954_final_masked.jpg]

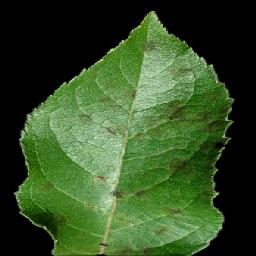

Supplement: Supplemental Information 2 [file peerj-cs-11-2543-s002.zip › Apple/Apple___Apple_scab/27338275-3c75-4bbf-8d74-7b90e9d224d3___FREC_Scab 3269_final_masked.jpg]

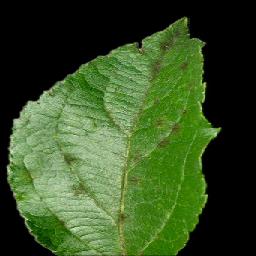

Supplement: Supplemental Information 2 [file peerj-cs-11-2543-s002.zip › Apple/Apple___Apple_scab/276ed34e-9987-4b38-b83b-8626504fc204___FREC_Scab 3050_final_masked.jpg]

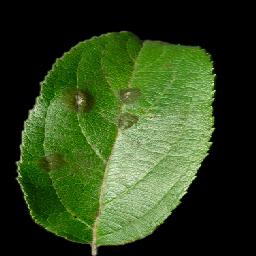

Supplement: Supplemental Information 2 [file peerj-cs-11-2543-s002.zip › Apple/Apple___Apple_scab/29ab8216-ec38-4efd-9c77-21068fa899a4___FREC_Scab 3241_final_masked.jpg]

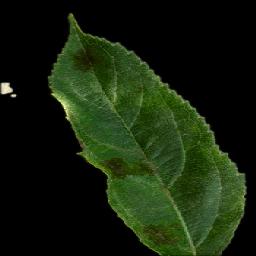

Supplement: Supplemental Information 2 [file peerj-cs-11-2543-s002.zip › Apple/Apple___Apple_scab/2a59761b-c91c-451c-8e13-d9dc3aff8d29___FREC_Scab 3503_final_masked.jpg]

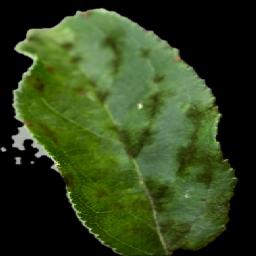

Supplement: Supplemental Information 2 [file peerj-cs-11-2543-s002.zip › Apple/Apple___Apple_scab/2a800c84-902e-4aa8-af13-4e020d6f17dd___FREC_Scab 3124_final_masked.jpg]

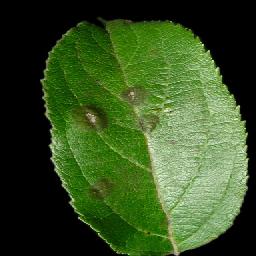

Supplement: Supplemental Information 2 [file peerj-cs-11-2543-s002.zip › Apple/Apple___Apple_scab/2a97b29d-8a63-47ab-bd5b-c14832a586c8___FREC_Scab 3242_final_masked.jpg]

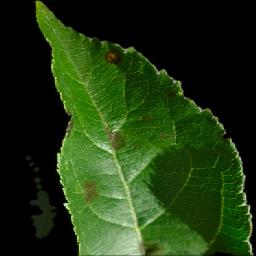

Supplement: Supplemental Information 2 [file peerj-cs-11-2543-s002.zip › Apple/Apple___Apple_scab/2ae81857-0615-4a6a-8d13-1ace0c62284d___FREC_Scab 2992_final_masked.jpg]

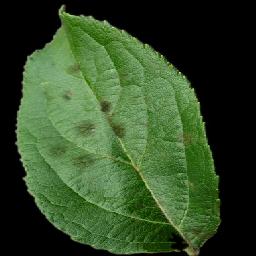

Supplement: Supplemental Information 2 [file peerj-cs-11-2543-s002.zip › Apple/Apple___Apple_scab/2bc018ba-3c49-4cf4-9411-a93b7b196f45___FREC_Scab 3326_final_masked.jpg]

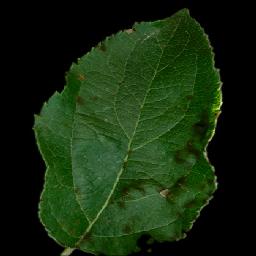

Supplement: Supplemental Information 2 [file peerj-cs-11-2543-s002.zip › Apple/Apple___Apple_scab/2bc50b52-0d7d-41a3-be8a-8d070341d87f___FREC_Scab 3476_final_masked.jpg]
